# COVID‐19 pneumonia and an indelible ground‐glass nodule

**DOI:** 10.1002/rcr2.751

**Published:** 2021-04-08

**Authors:** Sumitaka Yamanaka, Shinichiro Ota, Yukihiro Yoshida, Masaharu Shinkai

**Affiliations:** ^1^ Department of Thoracic Surgery Tokyo Shinagawa Hospital Tokyo Japan; ^2^ Department of Respirology Medicine Tokyo Shinagawa Hospital Tokyo Japan; ^3^ Department of Thoracic Surgery National Cancer Center Hospital Tokyo Japan

**Keywords:** COVID‐19, lung cancer, pneumonia, radiology and other imaging, thoracic surgery

## Abstract

When a chest computed tomography (CT) scan is performed in the diagnosis and treatment of coronavirus disease 2019 (COVID‐19) pneumonia, the possibility of lung neoplasm should be kept in mind if the ground‐glass nodule (GGN) shows features that are non‐specific for viral infection, such as solitary nature, relative roundness, well‐defined borders, and distance from the pleura.

## Graphical abstract

When a chest computed tomography (CT) scan is performed in the diagnosis and treatment of coronavirus disease 2019 (COVID‐19) pneumonia, the possibility of lung neoplasm should be kept in mind if the ground‐glass nodule (GGN) shows characteristics that are non‐specific for viral infection, such as solitary nature, relative roundness, well‐defined borders, and distance from the pleura.

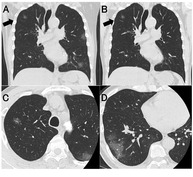

## Clinical Image

**Figure 1:**
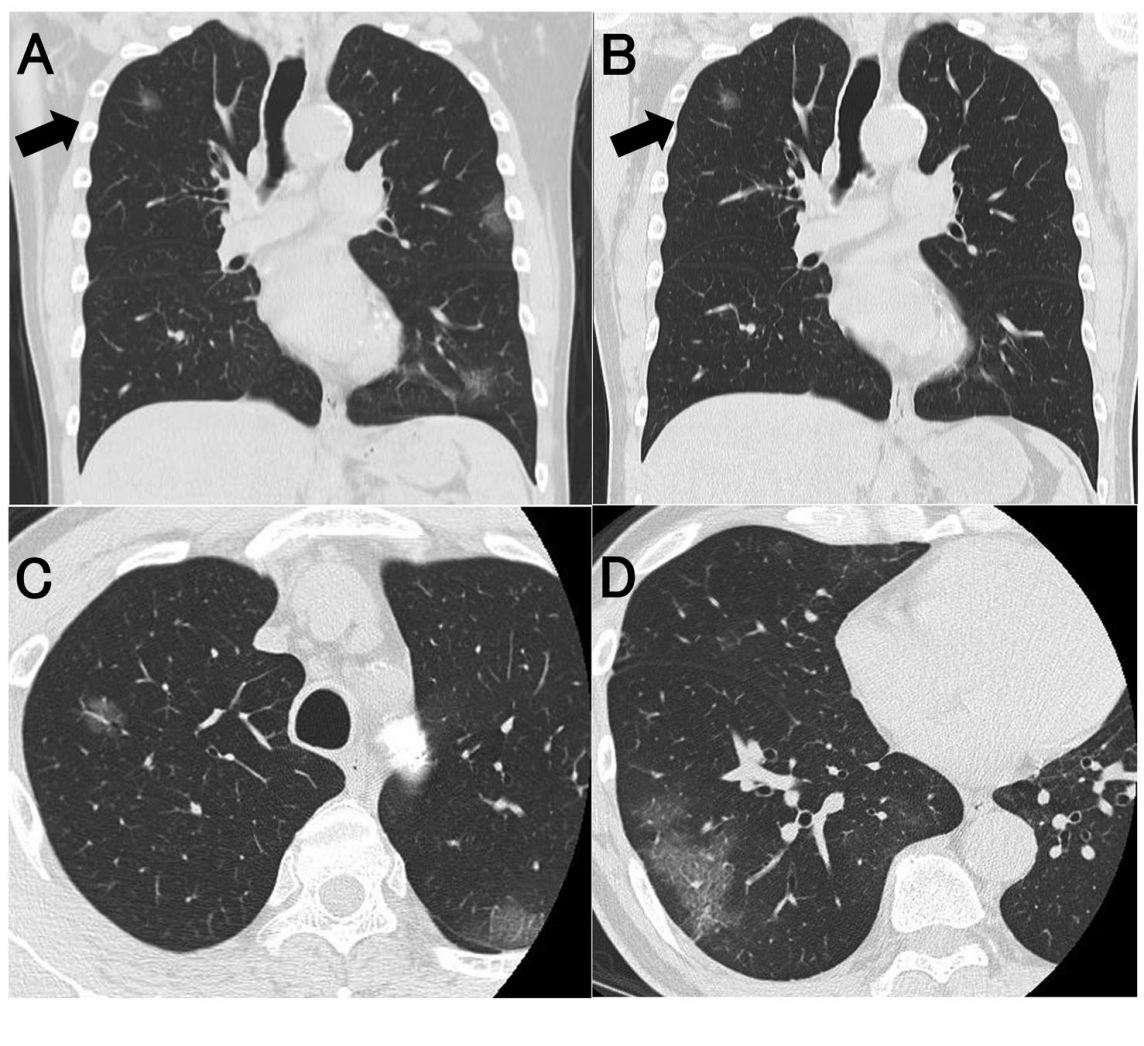
Coronavirus disease 2019 (COVID‐19) pneumonia and a primary lung adenocarcinoma. Chest computed tomography (CT) shows multiple patchy ground‐glass nodules, predominantly distributed in the peripheral lung parenchyma of the lower lobes in coronal section (A). Follow‐up CT three months after the first one shows that the ground‐glass nodule (GGN) (arrow) in the right upper lobe is still obvious (B). Thin‐slice CT of adenocarcinoma in situ (AIS) (C) and COVID‐19 pneumonia (D) in axial section. GGNs (D) are distributed in the periphery and subpleura, a typical finding in COVID‐19 pneumonia. In addition, peripheral GGNs (D) with superimposed intralobular reticulations resulting in a crazy‐paving pattern are shown [2]. A GGN (C), on the other hand, is lacking in these characteristic findings.

A 59‐year‐old man who complained of fever, general malaise, and olfactory and taste disorders was admitted to hospital five days after onset. Coronavirus disease 2019 (COVID‐19) pneumonia was diagnosed from the results of chest computed tomography (CT) and reverse transcription polymerase chain reaction test. Chest CT showed multiple patchy ground‐glass nodules (GGNs), mainly in the peripheral lung parenchyma beneath the pleura (Fig. 1A). A GGN in the right upper lobe seemed to show characteristics differing from other GGNs on chest CT, so follow‐up CT was scheduled after his recovery. He was discharged without sequelae after 19 days of treatment, and follow‐up CT was performed three months later (Fig. 1B). Only the GGN (Fig. 1B, arrow) in the right upper lobe remained, although the other pneumonia shadows had disappeared. Based on these imaging findings, primary pulmonary adenocarcinoma was suspected. Thoracic surgery was performed to obtain a definitive diagnosis, and adenocarcinoma in situ (AIS) was histopathologically diagnosed. Although follow‐up CT is not cost‐effective for all COVID‐19 pneumonia patients, the possibility of lung neoplasm should be kept in mind if the GGN shows characteristics non‐specific for viral infection, such as solitary nature, relative roundness, well‐defined borders, and distance from the pleura. If the clinician has such a suspicion, a follow‐up CT at low dose is recommended after one to three months according to the guidelines for pulmonary nodules with suspected infection [1].

### Disclosure Statement

Appropriate written informed consent was obtained for publication of this case report and accompanying images.

### Author Contribution Statement

All provided care for the patient. Sumitaka Yamanaka wrote the text and prepared the figures and Masaharu Shinkai edited the manuscript.
